# Clinical Determinants of 90-Day Mortality After Tracheostomy in Critically Ill Patients: A Multicenter Retrospective Cohort Study

**DOI:** 10.3390/medicina62061168

**Published:** 2026-06-16

**Authors:** Yakup Özgüngör, Hicret Yeniay, Burak Emre Gilik, Emre Karagöz, Mensure Çakırgöz, Sıla Seven

**Affiliations:** 1 Department of General Intensive Care Unit, İzmir City Hospital, İzmir 35530, Turkeydr.emrekaragoz@gmail.com (E.K.); silasevenn@gmail.com (S.S.); 2 Department of General Intensive Care, Balıkesir Atatürk City Hospital, Balıkesir 10185, Turkey

**Keywords:** tracheostomy, intensive care unit, 90-day mortality, APACHE II score, Charlson Comorbidity Index, prolonged mechanical ventilation

## Abstract

***Background and Objectives***: Tracheostomy is frequently performed in critically ill patients requiring prolonged invasive mechanical ventilation; however, factors associated with intermediate-term mortality after tracheostomy remain poorly characterized. This study aimed to identify clinical and procedural factors associated with 90-day all-cause mortality after tracheostomy in ICU patients. ***Materials and Methods***: This retrospective multicenter cohort study included 292 adult patients who underwent tracheostomy in two tertiary ICUs between 1 October 2023 and 1 June 2025. Demographic characteristics, admission diagnoses, comorbidities, clinical severity scores, procedural variables, microbiological culture results, and survival data were collected. Univariate analyses, multivariable binary logistic regression, Cox proportional hazards regression, receiver operating characteristic (ROC) analysis, and Kaplan–Meier survival analysis were performed. ***Results***: The overall 90-day all-cause mortality rate was 74.7%. Age, Charlson Comorbidity Index, and APACHE II score were significantly associated with 90-day mortality in univariate analyses, whereas tracheostomy timing and technique were not. In multivariable logistic regression analysis, the Charlson Comorbidity Index and APACHE II score were independently associated with mortality. Cox proportional hazards regression confirmed that both APACHE II score and Charlson Comorbidity Index were independently associated with mortality over time. ROC analysis demonstrated moderate discriminative performance for age, Charlson Comorbidity Index, and APACHE II score. ***Conclusions***: In critically ill patients undergoing tracheostomy, 90-day mortality was high and was driven primarily by acute illness severity and comorbidity burden rather than procedural characteristics. These findings support incorporating biological vulnerability, expected recovery potential, and goals-of-care discussions into tracheostomy decision-making.

## 1. Introduction

Tracheostomy is commonly performed in critically ill patients who require prolonged invasive mechanical ventilation or need persistent airway protection [1]. In the ICU setting, tracheostomy is substantially an elective or semi-elective procedure intended to facilitate ventilator weaning, improve secretion management, reduce sedation requirements, and enhance patient comfort [2]. This context differs fundamentally from emergency front-of-neck airway access procedures, which are performed in cannot-intubate/cannot-oxygenate scenarios and have distinct indications, urgency, and prognostic implications [3]. Therefore, the present study focuses specifically on ICU patients undergoing tracheostomy during the course of prolonged mechanical ventilation.

The advantages of tracheostomy comprise easier tube replacement once the tract is formed, improved speech, swallowing, and mobility, the ability to manage patients outside the intensive care setting, easier airway suctioning, improved oral hygiene, and greater airway stability during transfer or mobilization, thus providing enhanced overall patient comfort [4,5]. Tracheostomy-related complications vary substantially in both frequency and severity. Relatively common complications include minor bleeding, local stoma infection, tube obstruction, granulation tissue formation, and accidental decannulation, whereas rare but potentially catastrophic complications may entail tracheo-innominate artery fistula and mediastinitis. Therefore, complications should be interpreted according to their incidence, timing, and clinical severity rather than being presented as equivalent events [6].

There is no universally accepted optimal timing for tracheostomy. Although practice varies greatly among clinicians, a large number of them prefer performing tracheostomy between one and three weeks into the course of intubation. Conventionally, performing tracheostomy within the first 10 days of mechanical ventilation is not recommended since early tracheostomy has not been proven to provide significant benefit, and may lead to unnecessary surgical intervention and prolonged ventilation in patients who could otherwise be extubated [7].

Tracheostomy can be performed either by an open surgical or a percutaneous technique. The percutaneous approach offers several advantages over the surgical method. It can be performed at the bedside in the ICU, eliminating the need for an operating room, thereby reducing both procedure time and cost [8]. Moreover, some studies have reported lower rates of bleeding and infection with the percutaneously performed tracheostomy compared to the surgical technique [9]. Consequently, the percutaneous approach represents a practical option, particularly in ICU patients who require tracheostomy rather early during their clinical course. Nevertheless, it also poses certain disadvantages compared to the surgical approach, such as higher risk of anterior tracheal ring fractures, posterior wall perforation, and several specific relative contraindications [6].

The aim of this multicenter retrospective cohort study was to identify clinical and procedural factors associated with 90-day all-cause mortality after tracheostomy in critically ill patients, with particular emphasis on whether mortality was more closely associated with clinical severity of patients and comorbidity burden than with tracheostomy timing or technique.

## 2. Materials and Methods

This retrospective, observational, multicenter cohort study was approved by the Non-Interventional Ethics Committee of İzmir City Hospital (decision no: 2025/568; date: 5 November 2025). The study was conducted in two tertiary centers: İzmir City Hospital and Balıkesir Atatürk City Hospital. Throughout the manuscript, these centers are referred to as the İzmir center and the Balıkesir center, respectively. Since this was a retrospective, non-interventional study based on existing clinical records, it was not registered in a clinical trial registry. All adult patients who underwent either percutaneous or surgical tracheostomy between 1 October 2023 and 1 June 2025, were screened for eligibility.

Inclusion criteria were as follows:

- Patients aged 18 years old or above;
- Patients who required invasive mechanical ventilation for more than 7 days in the ICU;
- Patients who underwent either percutaneous or surgical tracheostomy during the study period.

Exclusion criteria were as follows:

- Patients admitted to the ICU with a pre-existing tracheostomy;
- Patients who underwent either prophylactic or permanent tracheostomy after otorhinolaryngologic surgery due to malignancy.

Patients were identified via the hospital’s electronic medical record system by selecting records documented as having undergone a tracheostomy procedure. The following data were collected: age, sex, APACHE II score, Charlson Comorbidity Index, primary reason for ICU admission, recent history of cardiopulmonary resuscitation prior to tracheostomy, renal replacement therapy requirement during ICU stay, duration of endotracheal intubation prior to tracheostomy, follow-up duration after tracheostomy, ICU and hospital length of stay, ICU discharge status, 90-day post-tracheostomy survival status, and microbiological culture results obtained during both intubation and tracheostomy periods. Time to tracheostomy was analyzed as a continuous variable, defined as the number of days from initiation of invasive mechanical ventilation to tracheostomy. No primary dichotomization into early and late tracheostomy was used in the main analysis. In the participating centers, APACHE II score is calculated within the first 48 h after ICU admission and is routinely updated at 7-day intervals throughout the ICU stay. For the purposes of this study, the most up-to-date/recently registered APACHE II score prior to the procedure was utilized in the analysis rather than only the initial admission APACHE II score. Deep tracheal aspirate culture positivity pre- and post-tracheostomy were recorded as microbiological data. Given the fact that standardized clinical, radiological, and microbiological adjudication of colonization, ventilator-associated tracheobronchitis, and ventilator-associated pneumonia was not consistently available in the retrospective records, culture positivity was interpreted only as a descriptive microbiological variable rather than as a definitive marker of lower respiratory tract infection. In both centers, tracheostomy was usually considered for patients with ongoing invasive ventilatory support needs, difficult weaning, or persistent airway protection requirements who were deemed high-risk for prolonged ICU or post-ICU care. Tracheostomy was not intended as an end-of-life procedure; however, due to the retrospective nature of the study, individual physician–family deliberations and goals-of-care discussions could not be standardized. Percutaneous tracheostomy was performed at the bedside in line with each institutions’ routinely favored practice. Bronchoscopic guidance was not used as a standardized routine practice in all percutaneous tracheostomies; its use depended on operator preference, patient anatomy, and each institutions’ procedural circumstances. Discharge-related outcomes were obtained from the electronic medical records. However, decannulation status, time to decannulation, liberation from mechanical ventilation, and functional recovery were not systematically recorded across both centers. Therefore, these outcomes were not included in the primary analysis.

In both centers, ventilator liberation was generally attempted by taking daily clinical-readiness assessment, which comprises improvement in oxygenation, hemodynamic stability, reduced sedative requirement, and adequate neurological status, into consideration. Spontaneous breathing trials were attempted when clinically feasible; however, a uniform written SBT protocol was not implemented across both centers during the study period. Due to the fact that individual SBT results, number of failed liberation attempts, and the specific physiological reasons for weaning failure were not consistently documented in the retrospective records, these variables could not be analyzed quantitatively.

The primary outcome was 90-day all-cause mortality after tracheostomy, defined as death from any cause occurring within 90 days from the date of tracheostomy. The date of tracheostomy was determined to be the starting point for follow-up. Mortality was not restricted to tracheostomy-related death.

Statistical analyses were performed using IBM SPSS Statistics for Windows, Version 26.0 (IBM Corp., Armonk, NY, USA). Categorical variables were presented as numbers and percentages. The distribution of continuous variables was assessed using the Shapiro–Wilk test. Normally distributed variables were expressed as mean ± standard deviation, whereas non-normally distributed variables were summarized as median and interquartile range (IQR). Chi-square or Fisher’s exact tests were used for categorical comparisons, and Student’s *t*-test or the Mann–Whitney U test was used for continuous variables as appropriate. The primary analysis was performed in the overall cohort according to 90-day all-cause mortality status. Inter-center comparisons were deemed secondary and supportive since the principal aim of the study was to evaluate factors associated with mortality rather than to compare institutional performance.

Variables associated with 90-day mortality in univariate analyses at a threshold of *p* < 0.05 were considered as candidate variables for multivariable logistic regression. The variables meeting this criterion were age, admission diagnosis, Charlson Comorbidity Index, and APACHE II score. Prior to model construction, candidate variables were further assessed for clinical interpretability, category size, and potential statistical instability. Admission diagnosis was not entered into the final multivariable model because it consisted of broad and clinically heterogeneous categories and several subgroups had very small cell counts, including oncological, nephrological, and hepatological admissions. In particular, the hepatological subgroup had no survivors, which could lead to unstable estimates or quasi-complete separation in logistic regression. Age was also not entered simultaneously with APACHE II and Charlson Comorbidity Index since it is already incorporated into both composite scores and was evaluated separately in the ROC analysis. Thereby, the final parsimonious multivariable logistic regression model included APACHE II score and Charlson Comorbidity Index.

In order to evaluate predictors of mortality over time, multivariable Cox proportional hazards regression analysis was performed. Hazard ratios (HRs) with 95% CIs were calculated. Bootstrap validation with 1000 resamples was applied to evaluate the stability of the logistic and Cox regression models. Survival probabilities were estimated using Kaplan–Meier survival analysis, and between-group differences were compared using the log-rank test.

Since clinically relevant inter-center heterogeneity was observed, formal interaction analyses were performed to evaluate whether the associations of the main mortality predictors differed between centers. The center variable was entered into the logistic regression model together with centered APACHE II score and centered Charlson Comorbidity Index. Interaction terms were then created for center × APACHE II score and center × Charlson Comorbidity Index and evaluated in separate multivariable logistic regression models using the enter method. A statistically significant interaction term was interpreted as evidence which points towards the association between the corresponding predictor and 90-day outcome differed by center.

A two-sided *p*-value < 0.05 was considered statistically significant.

## 3. Results

The study comprised 292 adult ICU patients who underwent tracheostomy during the study period. The overall 90-day all-cause mortality rate was 74.7% (218/292). Baseline categorical characteristics according to 90-day mortality status are presented in Table 1. ICU outcome and hospital discharge status are reported separately as descriptive post-tracheostomy discharge outcomes in Table 2. Among baseline categorical variables, on-admission diagnosis was significantly associated with 90-day mortality (*p* = 0.002). Diagnosis-specific 90-day mortality rates were 82.1% for pulmonary, 33.3% for oncological, 81.5% for cardiological, 73.3% for neurological, 77.8% for nephrological, 100.0% for hepatological, and 51.4% for trauma admissions, respectively. Sex, post-CPR status, renal replacement therapy requirement, and tracheostomy technique were not significantly associated with 90-day mortality. ICU outcome and hospital discharge status differed markedly between survivors and non-survivors; however, these variables were considered outcome-related measures and were interpreted descriptively rather than as baseline predictors.

Continuous clinical variables according to 90-day mortality status are summarized in Table 3. Age, Charlson Comorbidity Index, and APACHE II score were remarkably higher in non-survivors than in survivors (all *p* < 0.001). In contrast, the duration of mechanical ventilation prior to tracheostomy did not differ notably between the two groups (*p* = 0.698). Follow-up duration after tracheostomy, ICU length of stay, and hospital length of stay were longer among survivors; yet, these variables were considered outcome-related measures and were therefore interpreted descriptively rather than as baseline predictors of 90-day mortality.

Renal replacement therapy requirement was not significantly associated with 90-day mortality (*p* = 0.205). Similarly, tracheostomy technique was not associated with mortality; no significant difference was observed between patients undergoing percutaneous versus surgical tracheostomy (*p* = 0.796).

Deep tracheal aspirate culture positivity was observed in 216 patients (74.0%) pre-tracheostomy and in 218 patients (74.7%) post-tracheostomy. Prior to tracheostomy, the most frequently isolated microorganisms were *Acinetobacter* spp. (n = 55, 18.8%), *Klebsiella* spp. (n = 49, 16.8%), *Pseudomonas* spp. (n = 31, 10.6%), and MRSA (n = 26, 8.9%). After tracheostomy, *Acinetobacter* spp. remained the most common isolate (n = 78, 26.7%), followed by *Klebsiella* spp. (n = 61, 20.9%) and *Pseudomonas* spp. (n = 47, 16.1%). Neither pre-tracheostomy culture positivity (*p* = 0.404) nor post-tracheostomy culture positivity (*p* = 0.144) was significantly associated with 90-day mortality. Since culture positivity alone cannot distinguish airway colonization from ventilator-associated tracheobronchitis or ventilator-associated pneumonia, these findings were interpreted descriptively and were not included in the multivariable mortality model.

In univariate analyses of continuous variables, age, Charlson Comorbidity Index, and APACHE II score were significantly associated with 90-day mortality (all *p* < 0.001; whereas duration of mechanical ventilation prior to tracheostomy was not significantly associated with mortality (*p* = 0.698).

In order to appraise potential statistical overlap between age and the composite prognostic indices, Spearman correlation analysis was performed. Age exhibited a significant positive correlation with the Charlson Comorbidity Index (Spearman’s rho = 0.657, *p* < 0.001; n = 292) and a weaker but still significant positive correlation with APACHE II score (Spearman’s rho = 0.335, *p* < 0.001; n = 291). Hence, age was not entered simultaneously with APACHE II score and Charlson Comorbidity Index in the final multivariable logistic regression model in order to reduce redundancy and preserve model parsimony; instead, its discriminative performance was evaluated separately in ROC analysis.

In multivariable logistic regression analysis, the Charlson Comorbidity Index and APACHE II score were independently associated with 90-day mortality (Table 4). The overall model was statistically significant (omnibus test: χ^2^ = 33.043, *p* < 0.001), with Cox and Snell R^2^ = 0.107 and Nagelkerke R^2^ = 0.159. Although model calibration was acceptable according to the Hosmer–Lemeshow goodness-of-fit test (χ^2^ = 8.842, *p* = 0.356), the relatively low Nagelkerke R^2^ indicates limited explanatory capacity.

Each one-point increase in the Charlson Comorbidity Index was associated with approximately a 17.8% increase in the odds of 90-day mortality (OR = 1.178, 95% CI: 1.053–1.319; *p* = 0.004). Similarly, each one-point increase in APACHE II score was associated with approximately a 7.3% increase in the odds of mortality (OR = 1.073, 95% CI: 1.032–1.115; *p* < 0.001). Bootstrap validation confirmed the stability of these associations, with bootstrap *p*-values of 0.008 for the Charlson Comorbidity Index and 0.001 for the APACHE II score.

**Figure 1:**
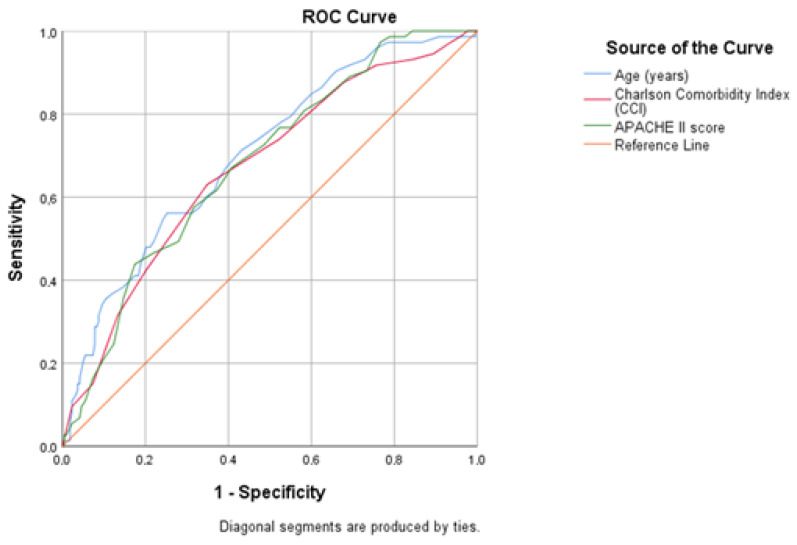
Receiver operating characteristic (ROC) curves of the Charlson Comorbidity Index and APACHE II score for predicting 90-day mortality.

ROC analysis demonstrated moderate discriminative performance for age, Charlson Comorbidity Index, and APACHE II score in predicting 90-day mortality. The AUC for age was 0.706 (95% CI: 0.639–0.773, *p* < 0.001), the AUC for the Charlson Comorbidity Index was 0.671 (95% CI: 0.599–0.742, *p* < 0.001), and the AUC for the APACHE II score was 0.681 (95% CI: 0.613–0.748, *p* < 0.001) (Figure 1).

In Cox proportional hazards regression analysis, the model including APACHE II score and Charlson Comorbidity Index was statistically significant overall (chi-square = 47.10, *p* < 0.001). Both variables were independently associated with mortality over time. Each one-point increase in APACHE II score was associated with a 3.9% increase in the hazard of death (HR = 1.039; 95% CI: 1.022–1.056; *p* < 0.001), and each one-point increase in the Charlson Comorbidity Index was associated with an 8.8% increase in the hazard of death (HR = 1.088; 95% CI: 1.039–1.140; *p* < 0.001). Bootstrap validation with 1000 resamples confirmed the stability of these associations.

**Figure 2:**
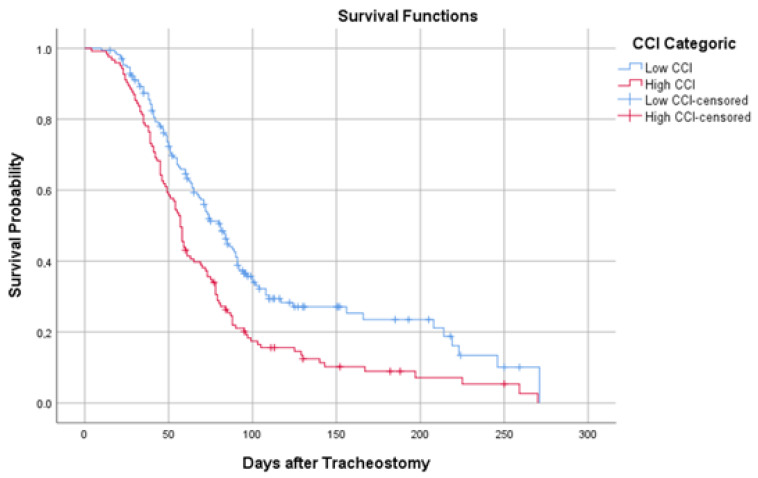
Kaplan–Meier survival curves according to Charlson Comorbidity Index category.

Kaplan–Meier survival analysis demonstrated that patients with a higher Charlson Comorbidity Index (CCI ≥ 5) had notably lower survival probabilities than those with lower Charlson scores (log-rank χ^2^ = 14.18, *p* < 0.001). The survival curves diverged early and remained separated throughout follow-up (Figure 2).

Secondary inter-center comparisons are presented in Appendix A (Table A1 and Table A2). Concisely, 192 patients were treated at the İzmir center and 100 at the Balıkesir center. The centers differed in on-admission diagnosis distribution, tracheostomy technique, Charlson Comorbidity Index, and time to tracheostomy; yet, 90-day mortality did not differ markedly between centers. Given that tracheostomy timing was not randomized, this inter-center variation may reflect differences in case-mix, patient selection, institution-dependent workflow, and institutional practice style rather than a causal effect of timing itself.

Due to the fact that substantial inter-center heterogeneity was observed in admission diagnosis distribution, tracheostomy technique, Charlson Comorbidity Index, and time to tracheostomy, formal center-by-predictor interaction analyses were performed. The center × APACHE II interaction term was statistically significant (B = −0.135, OR = 0.874, *p* = 0.022), suggesting that the association between APACHE II score and 90-day outcome differed between centers. In contrast, the center × Charlson Comorbidity Index interaction term was not statistically significant (B = −0.197, OR = 0.821, *p* = 0.206), indicating no clear evidence that the association between comorbidity burden and 90-day outcome was modified by center.

## 4. Discussion

The present multicenter cohort study investigated clinical and procedural determinants of 90-day all-cause mortality in critically ill patients undergoing tracheostomy after prolonged mechanical ventilation. The principal findings were as follows: 90-day mortality was high; APACHE II score and Charlson Comorbidity Index were independently associated with mortality in both logistic and Cox regression analyses, whereas procedural characteristics, including tracheostomy timing and technique, were not significantly associated with 90-day survival. These findings suggest that prognosis after tracheostomy is driven primarily by acute physiological severity and pre-existing comorbidity burden rather than procedural factors alone.

The 90-day mortality rate observed in the present cohort was high; however, it should be interpreted in the context of the specific population studied rather than as a direct consequence of the tracheostomy procedure itself. Previous studies have reported substantial variability in mortality after tracheostomy, depending on ICU type, case-mix, follow-up duration, and the severity of underlying critical illness. In a Turkish medical ICU cohort, Haşimoğlu et al. reported an ICU mortality rate of 82.4% among patients undergoing tracheostomy, and attributed this high mortality partly to advanced age, comorbidity burden, primary admission diagnosis, prolonged ICU stay, recurrent infections, and ICU-related complications [1]. In contrast, a neurocritical care cohort reported lower in-hospital mortality of 41.8% despite perpetual poor 3-month functional outcomes, with mRS 6 observed in 63.6% of patients at 3 months [10]. More recently, Jang et al. evaluated tracheostomized patients requiring prolonged mechanical ventilation for more than 21 days and reported cumulative mortality rates of 55.9%, 62.5%, and 66.7% at 3, 6, and 12 months, respectively [11]. These findings suggest that mortality after tracheostomy is highly dependent on the underlying clinical phenotype. Thus, the high mortality rate in our cohort likely reflects the combined effects of prolonged critical illness, high comorbidity burden, diagnosis distribution on admission, and institutional case-mix rather than procedural failure or mortality attributable to tracheostomy alone.

The main contribution of this study is the clarification of prognostic priorities in a multicenter population of tracheostomized ICU patients. APACHE II score and the Charlson Comorbidity Index are well established prognostic tools; hence, their association with mortality is not unprecedented. However, our findings demonstrate that even in the specific clinical context of tracheostomy, general markers of acute illness severity and comorbidity burden remain more illuminative than procedural variables, such as timing and technique. This provides practical insight for clinicians: when considering tracheostomy, prognosis should be concluded primarily by appraising biological vulnerability and recovery potential rather than the procedure itself.

The association between the APACHE II score and observed mortality in our study is expected and biologically plausible. The APACHE II scoring system has been extensively utilized in intensive care practice for decades and was originally developed to predict hospital mortality by integrating acute physiological derangements during the first 24 h of ICU admission with patients’ age and chronic health conditions. Therefore, the identification of APACHE II as an independent predictor of mortality in our cohort supports the clinical relevance of this association, and indicates that our findings are consistent with established pathophysiological determinants of outcomes in critically ill patients [12].

In contrast, the independent association between the Charlson Comorbidity Index (CCI) and 90-day mortality is particularly notable. The Charlson index was originally developed to identify the burden of comorbid disease and to estimate long-term prognosis, especially one-year and longer survival [13]. Our findings suggest that in critically ill patients undergoing tracheostomy, not only acute physiological derangement but also the pre-existing systemic disease burden plays a substantial role in determining intermediate-term survival. This observation indicates that outcomes following tracheostomy are influenced by the patient’s underlying biological reserve and comorbidity profile in addition to the severity of the acute illness. Consequently, these results support the notion that decision-maker clinicans, in terms of perfoming tracheostomy, should consider not only the current ventilatory status of the patient but also the overall comorbidity burden, which may predict post-ICU prognosis [14].

Even though APACHE II score and Charlson Comorbidity Index were independently associated with 90-day mortality, the multivariable logistic regression model had a relatively low explanatory capacity, as reflected by a Nagelkerke R^2^ of 0.159. Thus, these variables should not be interpreted as comprehensive individual-level predictors of mortality after tracheostomy. Rather, they represent markers of increased biological vulnerability within a broader and multifactorial clinical context. An immense proportion of mortality risk likely remains attributable to unmeasured or incompletely captured factors, including frailty, nutritional status, sarcopenia, evolving organ dysfunction after tracheostomy, pulmonary rehabilitation competence, quality of post-ICU care, and access to post-discharge healthcare services. Accordingly, the model should be viewed as an explanatory and prognostic framework rather than a stand-alone clinical prediction tool.

Age was significantly associated with 90-day mortality in univariate analysis. However, it was not entered separately into the final multivariable logistic regression model since age is already incorporated into both APACHE II score and the Charlson Comorbidity Index. Including age alongside these composite scores could have resulted in statistical overlap and collinearity. Therefore, age was considered clinically relevant; however, its prognostic contribution was interpreted through the broader domains of acute illness severity and comorbidity burden rather than as an isolated independent predictor.

The ROC findings also require cautious clinical interpretation. Although age, APACHE II score, and Charlson Comorbidity Index were statistically associated with 90-day mortality, their discriminative performance was only moderate, with AUC values ranging from 0.671 to 0.706. Hence, these parameters should not be used as stand-alone tools to select patients for tracheostomy or deny the procedure. In particular, APACHE II score and Charlson Comorbidity Index may help frame prognostic discussions by identifying higher-risk patients; nevertheless, they are not sufficiently accurate predictors to function as procedural gatekeepers on individual level. Decision-making regarding tracheostomy should instead integrate acute illness severity, comorbidity burden, trajectory of organ dysfunction, probability of ventilator liberation, pulmonary rehabilitation competence, patient values, and goals-of-care discussions with families. Future studies with larger and more homogeneous cohorts may be warranted to further investigate whether combined models incorporating dynamic physiological variables, frailty, nutritional status, functional trajectory, and post-ICU care factors might provide better discrimination than APACHE II score or Charlson Comorbidity Index alone.

Another remarkable finding of our study is that the timing of tracheostomy was not associated with 90-day mortality. The debate regarding early versus late tracheostomy remains ongoing in the literature. [4,7,15]. Recent systematic reviews and meta-analyses have demonstrated that premature tracheostomy may reduce the duration of mechanical ventilation and the length of stay in the intensive care unit in some studies; however, its effect on mortality remains inconsistent. For instance, a meta-analysis published in 2021 reported that early tracheostomy emerged to be superior in terms of ventilator-associated pneumonia rates, ventilator days, and ICU length of stay, whereas it did not demonstrate a significant reduction in short-term all-cause mortality [15]. A systematic review published in 2024 also suggested that early tracheostomy may provide benefits in certain clinical outcomes; however, the authors emphasized that additional randomized controlled trials are still warranted to draw definitive conclusions [16]. A review published in 2025 similarly indicated that the evidence remains inconclusive and that interpretations—particularly regarding mortality outcomes—should be made with caution [17]. Our findings are consistent with this uncertain line of evidence in the literatüre; despite a substantial difference in the time to tracheostomy between the two centers, this variation was not reflected in 90-day mortality. This observation suggests that, in the general ICU population, mortality may be driven less by the timing of the procedure itself and more by the patients’ underlying biological vulnerability and the severity of the acute illness. This finding should be interpreted cautiously. The difference between centers may reflect institutional practice style, institution-dependent procedural workflow, availability of bedside percutaneous tracheostomy, case-mix, and patient selection rather than timing alone.

The indication for tracheostomy is central to interpreting these results. In our cohort, tracheostomy was generally performed on patients with persistent need for invasive ventilation, difficult weaning, or ongoing airway protection needs, and those who were considered candidates for prolonged ICU or post-ICU care. It was not intended as an end-of-life intervention. Nevertheless, the high mortality rate indicates that many patients had limited physiological reserve despite being considered appropriate candidates for tracheostomy. This reinforces the need for early and explicit goals-of-care discussions prior to the procedure. Our cohort represents a high-risk population receiving treatment in tertiary general ICUs, with significant acute illness severity and comorbidity burden. Hence, the findings should not be extensively applied to elective tracheostomy populations, lower-severity ICU cohorts, specialized neurocritical or trauma cohorts, long-term weaning units, or centers with different discharge and palliative care practices.

The high proportion of survivors transferred to palliative care units should also be taken into account when interpreting hospital length of stay and medium-term outcomes. In our setting, transfer to palliative care does not necessarily indicate end-of-life tracheostomy intent prior to the procedure; rather, it may reflect the need for prolonged airway care, chronic ventilatory support, nursing care, and transition planning after ICU discharge. Nevertheless, such transfers may have influenced post-ICU survival, discharge trajectories, and observed hospital stay durations.

The microbiological findings should be interpreted meticulously. Although deep tracheal aspirate culture positivity was frequent both pre- and post-tracheostomy, culture positivity alone does not distinguish airway colonization from ventilator-associated tracheobronchitis or ventilator-associated pneumonia. The predominance of Gram-negative organisms, particularly *Acinetobacter*, *Klebsiella*, and *Pseudomonas* species, is consistent with the microbiological profile expected in prolonged mechanically ventilated ICU patients. However, due to the fact that standardized clinical and radiological criteria for infection adjudication were not consistently available across both centers, we did not attempt to classify culture-positive episodes as definite infection. Correspondingly, the microbiological data were retained only as descriptive information and should not be interpreted as evidence that tracheostomy increased or decreased infectious complications.

Inter-center heterogeneity deserves careful consideration. The İzmir and Balıkesir centers differed substantially in admission diagnosis distribution, preferred tracheostomy technique, comorbidity burden, and especially time to tracheostomy. These differences likely reflect not only patient-level clinical variation but also institution-dependent workflow, local procedural availability, physician practice patterns, and differences in thresholds for proceeding to tracheostomy. Despite similar 90-day mortality across the centers, formal interaction analysis demonstrated that the association between APACHE II score and 90-day outcome differed by center, whereas the association between Charlson Comorbidity Index and 90-day outcome did not. This finding suggests that the prognostic behavior of acute illness severity may be more sensitive to institutional case-mix, timing of score assessment, and clinical trajectory prior to tracheostomy, whilst the effect of comorbidity burden appears more consistent across centers. Therefore, the pooled analysis should be interpreted carefully and should not be mistaken as implying that both centers followed identical clinical pathways.

This study possesses a few important strengths. First, the sample size is larger than those of numerous previous single-center studies, and the inclusion of two tertiary care centers provides broader real-world data than a single-center cohort; yet, generalizability remains limited due to geographic proximity and institutional practice patterns. Second, both centers operate general intensive care units rather than highly specialized units focusing exclusively on neurological, trauma, or respiratory subpopulations, which increases the applicability of our results to a broader population of critically ill patients. Third, although the tracheostomy literature has frequently focused on procedural factors such as timing and technique, relatively few studies have evaluated 90-day mortality in relation to both acute illness severity and comorbidity burden within a multicenter framework.

Several limitations should be acknowledged. The retrospective observational design precludes causal inference and introduces the possibility of selection and indication bias. The cohort only included patients who survived long enough to undergo tracheostomy, whereas it did not include patients who died before tracheostomy, recovered without requiring the procedure, or had prolonged mechanical ventilation without tracheostomy. Thus, this study cannot determine whether mortality differed from those of severity-matched non-tracheostomized ICU patients. The observed mortality should consequently be interpreted as mortality within a selected tracheostomized ICU population rather than mortality attributable to tracheostomy itself.

Furthermore, even though the core notion behind performing tracheostomy was identifiable via institutions’ practical habits and clinical records, specific indications were not consistently registered as structured variables in the electronic medical records. In particular, the relative contributions of prolonged invasive mechanical ventilation, difficult weaning, failed spontaneous breathing trials, failed extubation, impaired airway protection, secretion burden, and neurological status could not be quantified. Similarly, although ventilator liberation was attempted after assessing daily clinical readiness in both centers, a uniform written spontaneous breathing trial protocol was not implemented across the study centers, and individual SBT results, the number of failed liberation attempts, and the physiological reasons for weaning failure were not invariably available. These limitations restrict the ability of the study to explain why patients required prolonged ventilatory support prior to tracheostomy.

Residual confounding may also persist despite multivariable adjustment. Important variables such as frailty, nutritional status, sarcopenia, baseline functional status, pulmonary rehabilitation competence, evolving organ dysfunction after tracheostomy, quality of post-ICU care, and access to post-discharge healthcare services were not systematically available. Consistent with this limitation, the relatively low Nagelkerke R^2^ of the logistic regression model indicates that APACHE II score and Charlson Comorbidity Index explained only a limited proportion of the variance in 90-day mortality. Hence, these variables should be interpreted as markers of increased biological vulnerability rather than a comprehensive individual-level prediction model.

Another limitation concerns the diagnostic categories. Even though admission diagnosis was associated with mortality in univariate analysis, it could not be reliably modeled as an independent predictor since the diagnostic categories were broad, clinically heterogeneous, and unevenly distributed, with very small numbers in some subgroups. Diagnosis-related mortality patterns should therefore be interpreted primarily as descriptive evidence of case-mix rather than adjusted independent effects.

Inter-center heterogeneity should also be considered. Tracheostomy decision-making, discharge practices, infection control strategies, palliative care referral patterns, post-ICU care pathways, and timing thresholds may have differed between centers. Despite similar 90-day mortality across the two centers, immense inter-center differences in case-mix and time to tracheostomy, together with the significant center × APACHE II interaction, suggest that institutional practice patterns may have influenced patient selection and the prognostic behavior of acute illness severity. Moreover, both centers were geographically close tertiary hospitals, which further limits generalizability to other healthcare systems, long-term weaning units, specialized neurocritical or trauma ICUs, and centers with distinct post-ICU care structures.

Formal pre-tracheostomy palliative or end-of-life designations were not systematically documented. Therefore, their potential influence on tracheostomy decision-making and subsequent mortality could not be analyzed. In addition, although transfer to palliative care units in our setting does not necessarily indicate end-of-life intent, it may reflect prolonged airway care, chronic ventilatory support, nursing care, and transition planning. This discharge pathway may have influenced post-ICU survival, length of hospital stay, and medium-term outcome trajectories.

Microbiological data were also limited by the retrospective nature of the study. These data were based on deep tracheal aspirate culture positivity and were not adjudicated using standardized clinical, radiological, and microbiological criteria to distinguish airway colonization from ventilator-associated tracheobronchitis or ventilator-associated pneumonia. Correspondingly, culture positivity should be interpreted as a descriptive microbiological variable rather than a definitive infection outcome.

The study also focused primarily on mortality and could not fully evaluate patient-centered outcomes. Even though discharge-related outcomes were available, detailed outcomes such as decannulation success, time to decannulation, liberation from mechanical ventilation, functional recovery, speech and swallowing outcomes, neurological improvement, quality of life, and long-term dependence on institutional care were not systematically recorded across both centers. Only three clearly documented in-ICU decannulation events were identified; consequently, a reliable decannulation rate could not be calculated. Thus, the clinical benefit of tracheostomy beyond survival could not be fully assessed.

Finally, the findings should be externally evaluated in independent cohorts before being generalized to other ICU populations. Future prospective studies should include severity-matched non-tracheostomized comparator groups, standardized documentation of tracheostomy indications and weaning attempts, and patient-centered outcomes such as ventilator liberation, decannulation, functional recovery, post-discharge healthcare services, and quality of life.

From a clinical perspective, the decision to perform tracheostomy should not be based solely on procedural considerations such as timing or technique. Rather, decision-making should incorporate comorbidity burden, acute physiological severity, expected recovery potential, patient values, and goals-of-care discussions with families. In this setting, APACHE II score and Charlson Comorbidity Index may provide a useful framework for communicating prognosis, while remaining complementary to clinical judgment.

## 5. Conclusions

In conclusion, 90-day all-cause mortality was high among critically ill patients undergoing tracheostomy. Acute illness severity, reflected by APACHE II score, and comorbidity burden, reflected by the Charlson Comorbidity Index, were independently associated with mortality. Conversely, procedural characteristics such as tracheostomy timing and technique were not significantly associated with survival. These findings suggest that prognostic assessment after tracheostomy should emphasize the patient’s underlying biological vulnerability and severity of critical illness, while recognizing that APACHE II score and Charlson Comorbidity Index alone provide only moderate discrimination, and should not be used as the sole basis for tracheostomy-related decision-making. 

## Figures and Tables

**Table 1:** Baseline categorical characteristics according to 90-day mortality status (*n* = 292).

Variable	Non-Survivors *n* = 218	Survivors *n* = 74	Total *n* = 292	*p*-Value
**Sex**				**0.510**
Male	129 (59.2%)	47 (63.5%)	176 (60.3%)	
Female	89 (40.8%)	27 (36.5%)	116 (39.7%)	
**Reason for ICU admission**				**0.002 †**
Pulmonary	101 (46.3%)	22 (29.7%)	123 (42.1%)	
Oncological	2 (0.9%)	4 (5.4%)	6 (2.1%)	
Cardiological	22 (10.1%)	5 (6.8%)	27 (9.2%)	
Neurological	63 (28.9%)	23 (31.1%)	86 (29.5%)	
Nephrological	7 (3.2%)	2 (2.7%)	9 (3.1%)	
Hepatological	4 (1.8%)	0 (0.0%)	4 (1.4%)	
Trauma	19 (8.7%)	18 (24.3%)	37 (12.7%)	
**Post-CPR status**				**0.185**
No	170 (78.0%)	63 (85.1%)	233 (79.8%)	
Yes	48 (22.0%)	11 (14.9%)	59 (20.2%)	
**Renal replacement therapy**				**0.205 †**
None	190 (87.2%)	70 (94.6%)	260 (89.0%)	
Intermittent hemodialysis	24 (11.0%)	4 (5.4%)	28 (9.6%)	
Continuous renal replacement therapy	4 (1.8%)	0 (0.0%)	4 (1.4%)	
**Tracheostomy technique**				**0.796**
Percutaneous	192 (88.1%)	66 (89.2%)	258 (88.4%)	
Surgical	26 (11.9%)	8 (10.8%)	34 (11.6%)	

† Fisher’s exact test was used.

**Table 2:** Descriptive post-tracheostomy discharge outcomes.

ICU Discharge Status	Overall n = 292
Death	227 (77.7%)
Palliative care transfer	58 (19.9%)
Transfer to another ICU	7 (2.4%)

Data are presented descriptively. No inferential comparisons were performed because discharge disposition occurred after tracheostomy and was closely related to subsequent survival trajectories.

**Table 3:** Continuous clinical variables according to 90-day mortality status.

Variable	Non-Survivors (*n* = 218) Median (IQR)	Survivors (*n* = 74) Median (IQR)	*p*-Value
Age, years	71 (61–81)	60 (46–71)	<0.001
Charlson Comorbidity Index	5 (4–7)	4 (2–6)	<0.001
APACHE II score	25 (20–31)	21 (17–25)	<0.001
Duration of mechanical ventilation prior to tracheostomy, days	30 (18–49)	30 (18–61)	0.698
Follow-up duration after tracheostomy, days	23.5 (12–43)	70 (37–116)	*
ICU length of stay, days	52 (36–74)	91 (56–124)	*
Hospital length of stay, days	54 (39–76)	108 (85–150)	*

Data are presented as median (interquartile range, IQR). Between-group comparisons were performed using the Mann–Whitney U test because continuous variables were not normally distributed. * No inferential comparison was reported for follow-up duration after tracheostomy, ICU length of stay, and hospital length of stay because these variables are outcome-related measures and were interpreted descriptively rather than as baseline predictors of 90-day mortality.

**Table 4:** Multivariable logistic regression analysis for 90-day mortality.

Variable	*p*-Value	OR for 90-Day Mortality	95% CI
Charlson Comorbidity Index	0.004	1.178	1.053–1.319
APACHE II score	<0.001	1.073	1.032–1.115

## Data Availability

The data supporting the findings of this study are not publicly available due to privacy and ethical restrictions related to patient data. De-identified data may be available from the corresponding author upon reasonable request and with the permission of the institutional ethics committee.

## Abbreviations

The following abbreviations are used in this manuscript:

ICU	Intensive Care Unit
APACHE II	Acute Physiology and Chronic Health Evaluation II
CCI	Charlson Comorbidity Index
MV	Mechanical Ventilation
HR	Hazard Ratio
CI	Confidence Interval
ROC	Receiver Operating Characteristic
AUC	Area Under the Curve

## Appendix A

**Table A1:** Demographic and clinical characteristics of the study population (*n* = 292).

Variable	İzmir (*n* = 192)	Balıkesir (*n* = 100)	Total (*n* = 292)	*p* (Between Centers) **	*p* (Overall Population) ***
**Sex**				0.184	0.510
Male	121 (63.0%)	55 (55.0%)	176 (60.3%)		
Female	71 (37.0%)	45 (45.0%)	116 (39.7%)		
**Reason for ICU admission**				<0.001 †	0.002 †
Pulmonary	107 (55.7%)	16 (16.0%)	123 (42.1%)		
Oncological	5 (2.6%)	1 (1.0%)	6 (2.1%)		
Cardiological	13 (6.8%)	14 (14.0%)	27 (9.2%)		
Neurological	36 (18.8%)	50 (50.0%)	86 (29.5%)		
Nephrological	5 (2.6%)	4 (4.0%)	9 (3.1%)		
Hepatological	3 (1.6%)	1 (1.0%)	4 (1.4%)		
Trauma	23 (12.0%)	14 (14.0%)	37 (12.7%)		
**Post-CPR status**				0.325	0.185
No	150 (78.1%)	83 (83.0%)	233 (79.8%)		
Yes	42 (21.9%)	17 (17.0%)	59 (20.2%)		
**Dialysis**				0.073 †	0.205
None	176 (91.7%)	84 (84.0%)	260 (89.0%)		
IHD	13 (6.8%)	15 (15.0%)	28 (9.6%)		
CRRT	3 (1.6%)	1 (1.0%)	4 (1.4%)		
**Tracheostomy technique**				<0.001	0.796
Percutaneous	160 (83.3%)	98 (98.0%)	258 (88.4%)		
Surgical	32 (16.7%)	2 (2.0%)	34 (11.6%)		
**ICU outcome**				0.049	*
Death	159 (82.8%)	73 (73.0%)	232 (79.5%)		
Survived	33 (17.2%)	27 (27.0%)	60 (20.5%)		
**90-day mortality**				0.638	*
Death	145 (75.5%)	73 (73.0%)	218 (74.7%)		
Survived	47 (24.5%)	27 (27.0%)	74 (25.3%)		
**Discharge status**				0.071 †	*
Death	157 (81.8%)	70 (70.0%)	227 (77.7%)		
Palliative care	31 (16.1%)	27 (27.0%)	58 (19.9%)		
Transfer to another ICU	4 (2.1%)	3 (3.0%)	7 (2.4%)		

† Fisher’s exact test was used. * No statistical comparison was performed because the variable is directly related to the 90-day mortality outcome. ** The corresponding variable in each row was compared between the two centers. *** The corresponding variable was compared between survivors and non-survivors in the overall study population (n = 292) with respect to 90-day mortality.

**Table A2:** Comparison of continuous variables between İzmir and Balıkesir patients.

Variable	Izmir Median (IQR)	Balıkesir Median (IQR)	*p* (Between Centers)	*p* (for 90-Day Mortality in the Overall Population)
Age (years)	70 (57.5–78.8)	67 (54–78.8)	0.163	<0.001
Charlson Comorbidity Index	5 (3.25–8)	4 (3–6)	0.003	<0.001
APACHE II score	23 (17.25–30.75)	24 (20–29)	0.445	<0.001
Time to tracheostomy (days)	22 (10–31.75)	62 (38.5–90.25)	<0.001	0.698
Time after tracheostomy (days)	27 (12.25–58.75)	37 (19–66)	0.045	*
ICU length of stay (days)	55 (39–82)	62 (38.5–91)	0.175	*
Hospital length of stay (days)	58.5 (41–90)	72 (42–98)	0.181	*

Data are presented as median (interquartile range, IQR). Normality of continuous variables was assessed using the Shapiro–Wilk test, which demonstrated that none of the continuous variables followed a normal distribution. Therefore, between-group comparisons were performed using the Mann–Whitney U test. * Statistical comparison was not performed because the variable is directly related to the 90-day mortality outcome.
